# CRZ1 regulator and calcium cooperatively modulate holocellulases gene expression in *Trichoderma reesei* QM6a

**DOI:** 10.1590/1678-4685-GMB-2019-0244

**Published:** 2020-05-08

**Authors:** Leonardo Martins-Santana, Renato Graciano de Paula, Adriano Gomes Silva, Douglas Christian Borges Lopes, Roberto do Nascimento Silva, Rafael Silva-Rocha

**Affiliations:** 1 Universidade de São Paulo, Faculdade de Medicina de Ribeirão Preto, Departamento de Biologia Celular e Molecular e Bioagentes Patogênicos, Laboratório de Biologia Sistêmica e Sintética, Ribeirão Preto, SP, Brazil.; 2 Universidade de São Paulo, Faculdade de Medicina de Ribeirão Preto, Departamento de Bioquímica e Imunologia, Ribeirão Preto, SP, Brazil.; 3 Universidade Federal do Espírito Santo, Centro de Ciências da Saúde, Departamento de Ciências Fisiológicas, Vitória, ES, Brazil.

**Keywords:** Gene regulatory network, cellulase expression, transcriptional regulation, calcium sensing

## Abstract

*Trichoderma reesei* is the main filamentous fungus used in industry to produce cellulases. Here we investigated the role of CRZ1 and Ca^2^+signaling in the fungus *T. reesei QM6a* concerning holocellulases production. For this, we first searched for potential CRZ1 binding sites in promoter regions of key genes coding holocellulases, as well as transcriptional regulators and sugar and calcium transporters. Using a nearly constructed *T. reeseiAcrz1* strain, we demonstrated that most of the genes expected to be regulated by CRZ1 were affected in the mutant strain induced with sugarcane bagasse (SCB) and cellulose. In particular, our data demonstrate that Ca^2^+ acts synergistically with CRZ1 to modulate gene expression, but also exerts CRZ1-independent regulatory role in gene expression in *T. reesei,* highlighting the role of the major regulator Ca^2^+ on the signaling for holocellulases transcriptional control in the most part of cellulases genes here investigated. This work presents new evidence on the regulatory role of CRZ1 and Ca^2^+ sensing in the regulation of cellulolytic enzymes in *T. reesei,* evidencing significant and previously unknown function of this Ca^2^+sensing system in the control key transcriptional regulators (XYR1 and CRE1) and on the expression of genes related to sugar and Ca^2^+ transport.

## Introduction

Cellulases and hemicellulases are key enzymes for the production of second generation ethanol production worldwide (Prasad et *al.,* 2007). The promising utilization of agroindustrial residues as energy source has triggered a tremendous interest for engineering microorganisms for the low cost production of these enzymes aiming the conversion of complex plant biomass into fermentable sugars, which in turn could be used for ethanol production (Druzhinina and Kubicek, 2017; Seppälä *et al*., 2017; Martins-Santana *et al*., 2018). In this sense, the saprophyte organism *T. reesei* is one of the main filamentous fungi used in industry to produce these enzymes (Druzhinina and Kubicek, 2017). Despite the great potential of this fungus for high performance cellulase

and hemicellulase production, the regulatory network controlling this system is relevant for studying and not fully understood yet. Additionally, the comprehension of transcriptional networks that coordinate the transcription of holocellulases is one of the main foci to allow a viable enzyme production at industrial scale (Borin *et al*., 2018).

*T. reesei* possesses a great capability for enzyme secretion (Schmoll *et al*., 2016) but few cellulases and hemicellulases genes. Even harboring this limited gene repertoire, its regulation is highly precise and controlled at molecular level by external signals (Gupta *et al*., 2016). Several transcription factors (TFs) are now known for exerting modulation on the expression levels of holocellulases genes (Paula *et al*., 2018). In this sense, the regulatory network that encompasses cell wall deconstruction is under regulation of positive regulators, such as XYR1 (Stricker *et al*., 2006), ACEII (Aro *et al*., 2001), LAE1 (Seiboth *et al*., 2012), BglR (Nitta *et al*., 2012), VEL1 (Karimi *et al*., 2014), HAP 2/3/5 complex (Zeilinger *et al*., 2001) and other proteins which play a negative control in such process, as CRE1 (Portnoy *et al*., 2011), ACEI (Aro *et al*., 2003) and RCE1(Cao *et al*., 2017). Additionally, it is already known that xylanases genes may also be targets of regulation by TFs, such as Xpp1 (Derntl *et al*., 2015b) and SxlR (Liu *et al*., 2017).

The complex network that coordinates holocellulases expression in *T. reesei* is also under regulation of nutritional variability. Therefore, differential gene expression was discussed by Castro *et al*. (2014b) and Antoniêto *et al*. (2016) in studies that reported the influence of carbon source on holocellulases transcription. Robust techniques like RNA sequencing have already highlighted the importance of sugar availability on the regulation mediated by TFs in *T. reesei* (Castro *et al*., 2016), evidencing the growing need of elucidating deep layers of regulation in this fungus.

Nutritional requirements and pH stress conditions control are targets of research among a wide number of fungal species, such as *Neurospora crassa* (Virgilio *et al*., 2017), *Trichoderma harzianum* (Moreno-Mateos and Codo, 2007) and *T. reesei* (He *et al*., 2014). However, the influence of several stress conditions, such as the effect of ion concentration exposure n transcription, still remain poorly understood in the industrial workhorse *T. reesei*. Despite not completely elucidated, recent studies have demonstrated the balance mediated by signaling pathways to keep physiological homeostasis when *T. reesei* is exposed to Ca^2+^ stress through Calmodulin-Calcineurin downstream activation steps (Chen *et al*., 2016).

CRZ1 (Calcineurin-responsive zinc finger 1) is a key target of Calcineurin (CN) in fungal cells and is a well-conserved homologus protein in several fungi species (Zhang *et al*., 2013; Thewes 2014; Chow *et al*., 2017), being responsible for many coordinated cellular functions. In *Saccharomyces cerevisiae,* the deletion of this protein resulted in hypersensitivity to chloride and chitosan, as well as mating and transcriptional response to alkaline and stress conditions defects (Zakrzewska *et al*., 2005). In another *crz1* mutant yeast, *Candida albicans*, the knockout effect was correlated to changes on the pattern of sensitiveness to Ca^2+^, Mn^2+^ and Li^2+^, as well as in hypersensitiveness to azole molecules (Santos and de Larrinoa, 2005). The role of CRZ1 in filamentous fungi is already described for some species, and in this context, for the pathogenic and opportunistic *Aspergillus fumigatus, crz1* homologue *(crzA)* knockout resulted in virulence attenuation and developmental defects in both hyphal morphology and conidiation (Soriani *et al*., 2008). Effects of CRZ1 in filamentous fungi were also mentioned for *Cryptococcus neoformans*, where growth at temperatures higher than 39 °C was compromised in *crz1* knockout strains, as well as its absence resulted in sensitiveness to cell wall perturbing agents (Lev *et al*., 2012; Chow *et al*., 2017).

In *T. reesei,* the role of Ca^2+^-CaM/CN/CRZ1 pathway is poorly studied and established in comparison to other fungal species. Yet, it is already known that the Ca^2+^signaling pathway may exert effects on the modulation of holocellulases gene expression in the industrial strain of *T. reesei* RUT-C30 (Chen *et al*., 2016). Therefore, Chen *et al*. (2016) showed that the regulation of relevant industrial genes, such as *cbh1, eg1* and *xyr1* genes were negatively modulated in the absence of CRZ1 when calcium was added to a cellulose-supplemented medium.

Despite the evidence that CRZ1 and Ca^2+^ may influence holocellulases expression, these results were obtained when the industrial strain of *T. reesei* RUT-C30 was evaluated in different environmental conditions. Yet, this strain harbors a deletion of approximately 85 kilobases (kb) from the *T. reesei* genome which includes the *cre1* locus, resulting in phenotypes that are independent on CRE1-mediated influence (Seidl *et al*., 2008; Mello-de-Sousa *et al*., 2014).

The role of CRZ1 and Ca^2+^ have not been investigated in the wild type strain of *T. reesei* (QM6a), which harbors the full set of regulatory components in its genome. In order to elucidate this, we investigated the role of CRZ1/Ca^2+^ in a wild set genes coding for TFs, enzymes, and transporters related to holocellulose deconstruction. We were able to identify CRZ1 transcription factor binding sites (TFBS) in promoter regions of holocellulases differentially expressed genes after *T. reesei* QM6a exposure to SCB and cellulose as carbon source. Additionally, we reported the fundamental role of CRZ1 on transcriptional regulation of such genes, as well as demonstrating that Ca^2+^ can cooperatively with CRZ1, induce or repress the transcription of holocellulase genes. These findings bring evidence of Ca^2+^ as a pivotal ion in the control of stimuli regarding expression of sugar and calcium transporters in *T. reesei* QM6a cells in a CRZ1-independent but synergistic mechanism.

## Material and Methods

### Analysis of promoter sequences

For the identification of potential CRZ1-binding elements, sequences of 1kb upstream of the ATG from the Open Reading Frames (ORFs) of the promoter of TFs, holocellulases and transporter genes, as well as for homologs of calcium and sugar transporters were retrieved from the reference genome of *T. reesei.* Next, Position Weight Matrix (PWM) from CRZ1 of *S. cerevisiae* were obtained from YeTFaSCo (The Yeast Transcription Factor Specificity Compendium) collection (De Boer and Hughes, 2012) and used to calculate the scores in all promoters of *T. reesei.* Scores greater than zero were selected and normalized, revealing sequence with the higher probability of harboring a functional *cis*-regulatory element than a random motif. The normalization function was: Score_Normalize_d (Xij) = Score(Xij)/MaxScore, being X the set of sense and antisense upstream regulatory sequences; *i* the sequence index; *j* the initial position for score calculation; and MaxScore the maximum score possible of the CRZ1 TF PWM. Through histogram analyses of the normalized score, we established a threshold of 0.8, using a comparison between real sequences and a control dataset formed by random sequences. Finally, this threshold was used to map potential cis-regulatory elements for CRZ1 in the target of interest.

### Strains and media

*T. reesei* strain QM6a Δ*tmus53* Δ*pyr4* was used as wild type (WT) strain in this study and it was obtained from the Institute of Chemical Engineering (Viena University of Technology, Viena, Austria) (Derntl *et al*., 2015a). The strain was maintained in MEX medium (3% (w/v) malt extract and 2% (w/v) agar)) at 30 °C supplemented with 5 mM uridine for *pyr4* auxotrophic selection (Gruber *et al*., 1990). *T. reesei* QM6a Δ*tmus53* Δ*pyr4* wild type and Δ*crz1* (constructed as described below) strains were grown on MEX medium at 30 °C for 7-10 days until complete sporulation. For gene expression assays, approximately 10^6^ spores/mL from both strains were inoculated in Mandels Andreotti (MA) medium (Schmoll *et al*., 2009) containing 1% (w/v) sugarcane bagasse, cellulose (Avicel, Sigma-Aldrich) or glycerol as carbon source. For induction of gene expression in the presence of sugarcane bagasse, the substrate *in natura,* gently donated by Nardini Agroindustrial Ltd., Vista Alegre do Alto, São Paulo, Brazil, was previously prepared according to Souza *et al*. (2011). Mycelia from both strains (WT and Δ*crz1*) were pre-grown in MA medium supplemented with 1% (w/v) glycerol, in the presence and in the absence of 10 mM CaCl_2_, in orbital shaker at 30 °C for 24 h under 200 rpm. In these assays, MA medium presented a basal concentration of 2.6 mM Ca^2+^ to ensure the maintenance of fungal cellular viability and growth. The obtained mycelia were harvested and transferred to MA medium supplemented with 1% (w/v) sugarcane bagasse or cellulose (Avicel, Sigma-Aldrich) in orbital shaker at 200 rpm, 30 °C for 8 h. All experimental conditions described were performed with three biological replicates. The resulting mycelia were collected by filtration, frozen in liquid nitrogen and stored at -80 °C for RNA extraction procedures.

### Construction of crz1 deletion cassette and *T. reesei* genetic transformation

Deletion of *crz1* gene from *T. reesei* QM6a Δ*tmus53* Δ*pyr4*was performed as previously described (Schuster *et al*., 2012). For this, we designed a deletion cassette containing the *pyr4* gene (orotidine-5'-phosphate decarboxylase gene of *T. reesei - Trire_74020)* as auxotrophic selection marker. All sequences of this cassette are available at the *T. reesei* genome database (https://mycocosm.jgi.doe.gov/Trire2/Trire2.home.html) and the primers used in this study were designed to contain overlapping regions to the components sequence of the cassette (Table S1). The deletion cassette is composed by 1226 base pairs (bp) upstream to the 5’ *crz1* CDS flanking region *(Trire_36391)* and 1465 bp downstream to the *crz1* ORF 3’ flanking region. These two sequences flanks *pyr4* gene sequence, which consists of 1100 bp upstream and 1000 bp downstream to the *pyr4* CDS sequence in *T. reseei’s* genome. Individual fragments were amplified at 60°C with Phusion® High-Fidelity Polymerase (New England Biolabs) using *T. reesei* QM6a Δ*tmus53* Δ*pyr4* genomic DNA as template, according to the manufacturer’s instructions. The PCR fragments were purified using QIAquick PCR Purification Kit (Qiagen). The assembly of the components was performed through homologous recombination in *S. cerevisiae,* as previously reported (Colot *et al*., 2006; Gietz and Schiestl, 2007; Collopy *et al*., 2010). For this purpose, we used the pRS426 shuttle vector (Christianson *et al*., 1992) previously treated with EcoRI and*XhoI* enzymes (New England Biolabs). These

treatments resulted in the occurrence of cohesive ends correspondent to the external extremities in the primers used to obtain the 5’ and 3’ *pyr4* ORF flanker regions of the deletion cassette. Yeast transformation procedures were performed as described by Gietz and Schiestl (2007) in the yeast *S. cerevisiae* strain SC9721 *(MATα his3*Δ200 URA3-52 leu2Δ1 lys2Δ202 trp1Δ63) (Fungal Genetic Stock Centerwww.fgsc.net). The transformants were selected on YNB (0.7% (w/v) yeast nitrogen base (Sigma) supplemented with 2% glucose, 0.1 g/L lysine, 0.05 g/L histidine, 0.1 g/L leucine, and 0.1 g/L tryptophan in the absence of uracil.

To confirm the complete assembly of the cassette, genomic DNA of *S. cerevisiae* was extracted according to Goldman *et al*. (2003) and Sambrook and Russel (2001). Primers 5’Pcrz1 and 3’Tcrz1 (Table S1) were used to amplify the deletion cassette from the yeast genome using Phusion® High-Fidelity Polymerase (New England Biolabs) according to manufacturer’s instructions. The resultant amplicons were purified using QIAquick PCR Purification Kit (Qiagen) and were stored at -20 °C until *T. reesei* transformation. Transformation of *T. reesei* was performed using 10 µg of the linearized deletion cassette by protoplast fusion methodology (Gruber *et al*., 1990). Transformants were selected in MA medium plates supplemented with 1% (w/v) glucose and 2% (w/v) agar (Sigma-Aldrich). Selection was carried out in the absence of uridine. Genomic DNA was extracted to confirm the cassette integration (Vitikainen *et al*. (2010). To verify the correct orientation of cassette positioning in the positive transformants, it was performed two PCR reactions with primers 5’Pcrz1 Check1/3’pyr4 Check 1 and 5’pyr4 Check 2/3’T*crz1* Check 2 (Table S1, Figure S1 A-B), which are specific for *pyr4* annealing regions and for external annealing regions into promoter and terminators sequences. A PCR to confirm the deletion of the *crz1* from *T. reesei* genome was performed using the primers 5’*crz1* ORF and 3’crz1 ORF (Table S1, Figure S1 C). Null expression of *crz1* gene was also investigated with a quantitative PCR with total RNA using the primers PF *crz1* qRT-PCR and PR *crz1* qRT-PCR (Table S1, Figure S1 D).

### Gene expression analysis by Real-Time PCR

For gene expression analysis, 1 µg of total RNA of each culture condition was treated with DNAse I (Sigma-Aldrich). cDNA samples were synthesized using Maxima First Strand cDNA Synthesis kit (ThermoFisher Scientific) and posteriorly diluted 1:50 in DEPC water. For gene expression levels detection, SsoFast EvaGreen® Supermix (Bio-Rad) was used according to the manufacturer s instructions. A list of target genes whose expression were evaluated are available in Table S2. Reactions were carried out at 95 °C for 10 min, followed by 40 cycles at 95 °C for 10 s and 60 °C for 30 s in a Bio-Rad CFX96 Real-Time System coupled to a C1000 Thermal Cycler (BioRad). Expression of target genes was normalized by the β-actin endogenous transcript levels for each RNA prevenient from the culture conditions. For sugarcane bagasse and cellulose expression quantification (for both in presence or in the absence of 10 mM calcium chloride) the 2^-ΔΔCt^ method was employed (Livak and Schmittgen, 2001). The quantification was relative to transcript levels observed in the WT and *Δcrz1* strains grown in glycerol for 24 h (for both in the presence or in the absence of 10 mM calcium chloride).

### Enzymatic activity assays

The activity of β-glucosidases and β-xylosidases were measured as the capacity of hydrolyzing pNPG and pNPX substrates as previously described (Hideno et al., 2011; Lopes *et al*., 2012; Castro *et al*., 2014b). Endoglucanase activity was determined using carboxymethylcellulose (CMC) (Sigma-Aldrich) as substrate in microplates, in a protocol adapted from Xiao *et al*. (2005). We solubilized 1% (w/v) CMC in pH 4.8 sodium acetate buffer. After addition of 30 µL of culture supernatant, reactions were incubated for 30 min at 50 °C. After this time, 60 µL of 3,5-dinitrosalicylic acid (DNS) were added to the reaction and the mixture were submitted to incubation for 5 min at 95 °C (Miller, 1959). Absorbance at 540 nm was used for samples measurements. One enzyme unit was defined as the amount of enzyme capable of releasing 1 µmol of reducing sugar per minute. Endoxylanases activity was determined using xylan from beechwood (Sigma-Aldrich) solubilized in pH 5.0 100 mM sodium acetate buffer as substrate. Reactions were performed with 25 µL of the culture supernatant and 50 µL of substrate at 50 °C for 30 min. After incubation, we mixed 75 µL of DNS and heated the reactions at 95 °C for 5 min to visualize the effects of reduction of dinitrosalicylic acid. Absorbances were carried at 540 nm. One unit of enzyme was defined as the amount of enzyme capable of releasing 1 µmol of reducing sugar per minute (Castro *et al*., 2014a).

## Results

### Identification of CRZ1 binding motifs in *T. reesei* promoters

**Figure 1:**
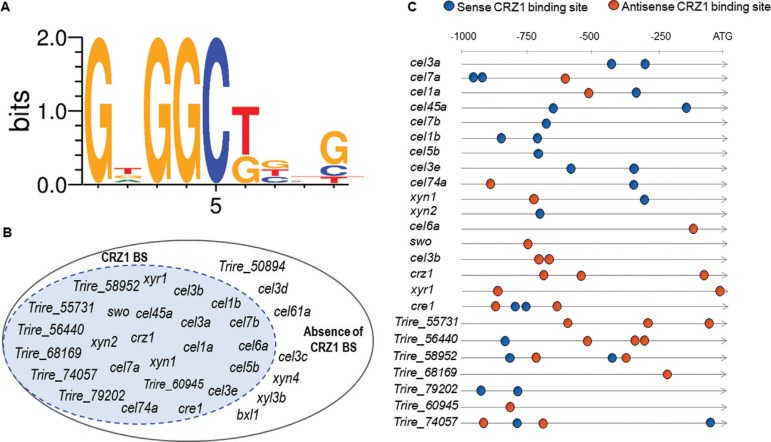
(A) DNA Consensus sequence of CRZ1 binding in *T. reesei* obtained through bioinformatics analysis. (B) Representative scheme of genes which harness CRZ1 BS (CRZ1 binding sites) at genomic level. (C) *in silico* Identification of CRZ1 BS in promoter regions of holocellulases and calcium and sugar transporter genes in *T. reesei.* Blue spheres indicate that those sequences (previously shown in 1A) have been found in sense orientation, and orange spheres represent the antisense sequences occurrence. The number of spheres represents the absolute frequency that these sequences were observed in bioinformatics analysis.

We first investigated the existence of putative binding sites for CRZ1 in *T. reesei* holocellulases and calcium and sugar transporter promoters using the defined sequence for *S. cerevisiae.* For this, we selected 30 genes related to biomass degradation in plant cell wall through proteins secreted by *T. reesei,* such as cellulases, hemicellulases and sugar transporters, as well as calcium transporters and TFs that play a significant role in this process. In this sense, we were able to identify potential cis-regulatory elements for CRZ1 in 24 of these sequences, as represented in Figure 1A-B. Interestingly, the major numbers of potential cis-regulatory elements for CRZ1 were observed in *cre1* and *cel7a* genes, as well as for *Trire_56440* and *Trire_58952* calcium transporter proteins promoters (Figure 1C). We also observed that the promoter of *crz1* gene harbors 3 potential cis-regulatory elements in antisense orientation, suggesting that CRZ1 plays autoregulation at transcriptional level (Figure 1C). Taken together, these results evidenced the existence of putative binding sites for CRZ1 in several genes related to gene regulation, biomass and calcium transport, highlighting the potential role of this TF in the regulation of these genes.

### CRZ1 and Ca^2^+ synergistically regulate TFs and cellobiohydrolase coding genes

**Figure 2:**
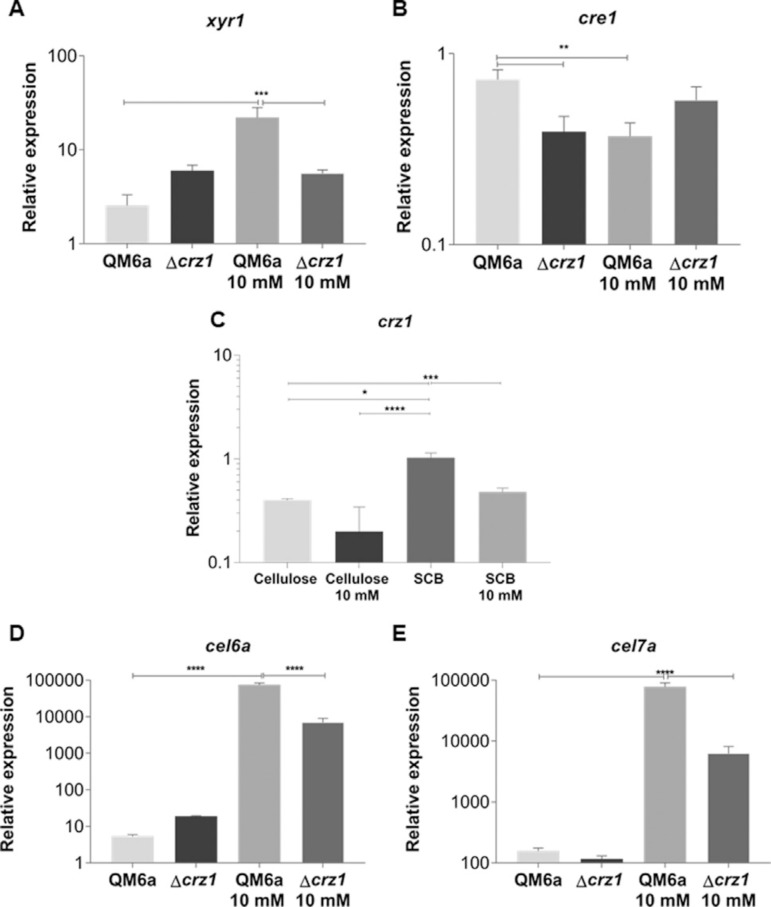
Analysis of differential expression of the transcription factors XYR1 (A), CRE1(B), and CRZ1 (C) of *T. reesei* and of cellobiohydrolases CEL6A (D) and CEL7a (E) from QM6a and Δcrz1 T. reesei strains after 8 h of growing in SCB and SCB supplemented with 10 mM Ca^2^+. CRZ1 expression in the presence of cellulose and the effect caused by calcium on this induction is also shown in panel C. Expression values are represented as log10 means of three biological replicates with standard deviation normalized by glycerol expression levels at the same condition. Statistical significance is represented as asterisks, considering p-value as <0.05 (*), <0.01 (**), <0.001 (***) and p<0.0001 (****).

Once we identified putative binding sites for CRZ1 at promoter regions of the genes of interest, we tried to understand how carbon source, as well as Ca^2+^ supplementation, could modulate the transcription of these genes in the wild type and Δcrz1 mutant strain. In this sense, we observed that under exposure to SCB, the expression of *xyr1* was positively modulated in the wild type strain of *T. reesei* by the supplementation of 10 mM Ca^2^+ to the medium (Figure 2A). However, this stimulatory effect was lost in the *T. reesei* Δcrz1 strain, indicating that this process was completely dependent on CRZ1. Additionally, the same effect was observed when Avicel was used as inducer (Figure S2). In the case of *cre1,* we observed only minor changes in the expression of this TF under all conditions tested, with a small but significant reduction in *its* expression in the wild type strain upon Ca^2^+ induction or between wild type and Δcrz1 strain without the exposure to this ion (Figure 2B). Yet, when its expression was assayed under Avicel exposure, no significant change was observed between any condition analyzed (Figure S2 B). It is worth to notice that while putative CRZ1 binding sites were identified at both *xyr1* and *cre1* promoters (Figure 1C), only the former seems to be significantly regulated by this TF under the conditions analyzed here.

Since we identified putative CRZ1 binding sites in *crz1* promoter, we were also interested in investigate how CRZ1-mediated autoregulation in *T. reesei* was related to carbon source availability. In this sense, Figure 2C shows the result only for wild type strain of *T. reesei* with or without Ca^2+^ exposure. As shown by the figure, under SCB induction, the expression levels of *crz1* were higher than those obtained after cellulose induction. In addition, after supplementation of the medium with 10 mM Ca^2+^, the expression of this gene decreased in the presence of SCB, suggesting that Ca^2^+ may exert a modulatory effect on CRZ1 autoregulation.

Since we observed that *xyr1* expression was induced by Ca^2+^ in a CRZ1 dependent manner, we next evaluated the role of these two components in the expression of *cel6a* and *cel7a,* the two major cellobiohydrolases from *T. reesei*. We found that Ca^2+^ supplementation resulted in a significant increase in *cel6a* and *cel7a* induction both under exposure to SCB (Figure 2D-E) for *cel6a* in Avicel (Figure S2 C-D). Additionally, deletion of crz1 resulted in a significant reduction in this stimulatory effect, even though this was not completely abolished. Taken together, these results indicated that CRZ1 and Ca^2+^ synergistically control the expression of celobiohydrolases coding genes under SCB and Avicel exposure, and highlight that an additional Ca^2+^-dependent and CRZ1-synergistic mechanism could take part in this process.

### Other cellulases genes are also key of regulation by CRZ1 and Ca^2^+

**Figure 3:**
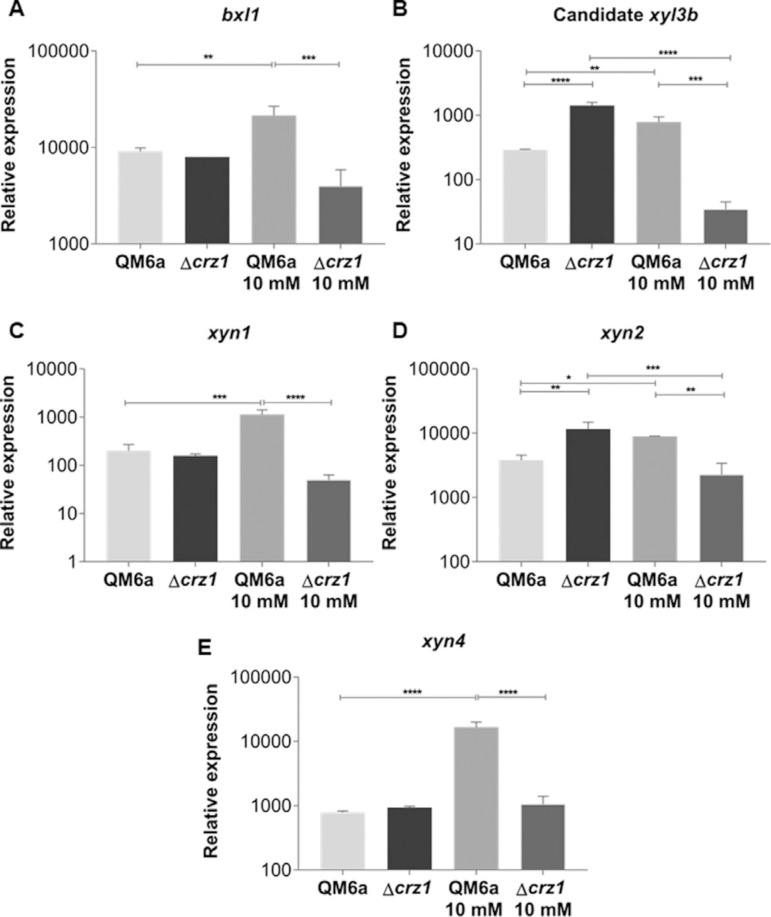
Relative expression of the β-xylosidase 1 and (A) the candidate *xyl3b* xylosidase (B) and of the endoxylanases *xyn1* (C), *xyn2* (D) and *xyn4* (E) genes from QM6a and Δcrz1 T. reesei strains after 8 h of induction SCB and SCB supplemented with 10 mM Ca^2^+. Expression values are represented as log10 means of three biological replicates with standard deviation normalized by glycerol expression levels at the same condition. Statistical significance is represented as asterisks, considering p-value as <0.05 (*), <0.01 (**), <0.001 (***) and p<0.0001 (****).

Since we observed that CRZ1 is capable to modulate *xyr1* expression levels, we next investigated how the expression of xylanase coding genes was modulated as a result of such effect mediated by CRZ1. Thus, under exposure to SCB, we observed a significant increase on the expression of these genes induced by the simple supplementation with Ca^2+^ (Figure 3A-E). Additionally, the deletion of *crz1* was not able to result in differences on the expression of these genes, but we detected a loss in their expression in the *T. reesei* Δcrz1 strain after Ca^2^+ supplementation. Taking in advantage of these results, our findings suggest that the regulation of xylanase genes under SCB exposure is modulated by Ca^2^+ in a synergistically CRZ1-dependent mechanism, highlighting the relevance of this TF on a complete background concerning gene regulation in the wild type strain of this fungus. Interestingly, no effects were observed when Avicel was used as inducer, since none differential expression was detected under cellulose exposure (Figure S3 A-D).

**Figure 4:**
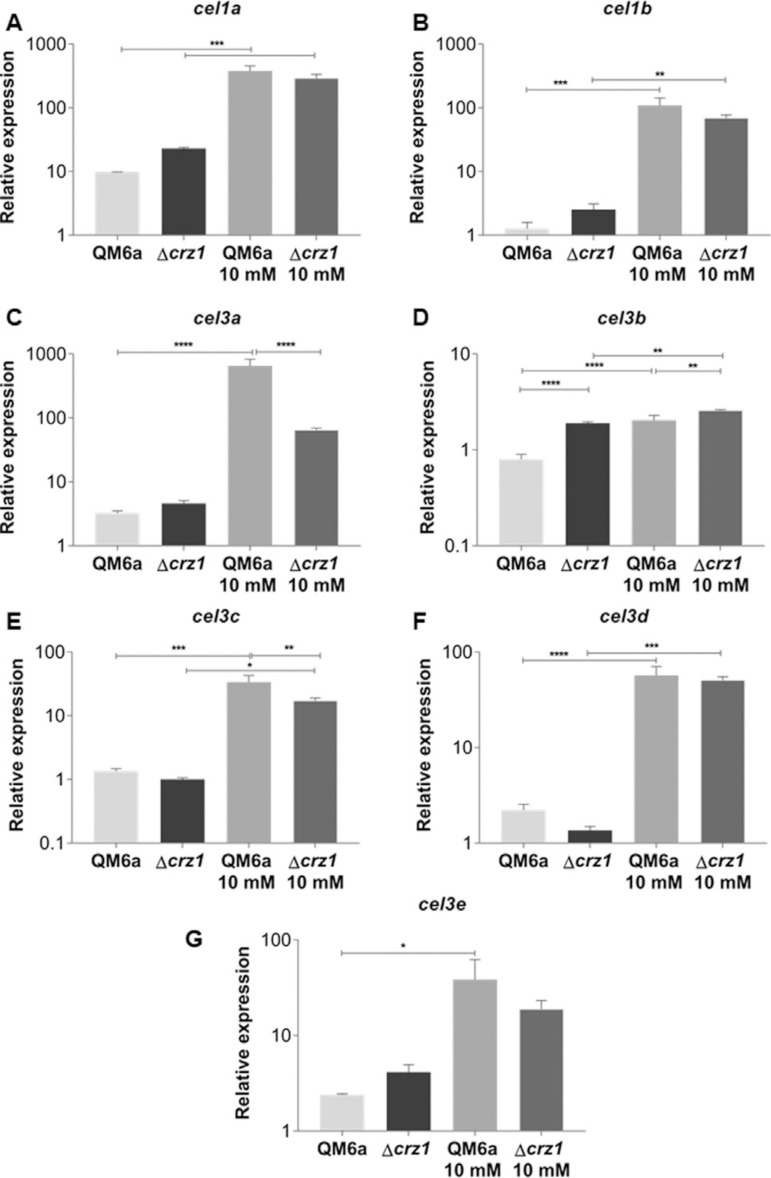
qRT-PCR results for differential expression analysis of the β-glucosidases genes of *T. reesei* in the QM6a and Δcrz1 strains after 8 h of growing in SCB supplemented or not with 10 mM Ca^2^+. Expression values are represented as log_10_ means of three biological replicates with standard deviation normalized by glycerol expression levels at the same condition. Statistical significance is represented as asterisks, considering p-value as <0.05 (*), <0.01 (**), <0.001 (***) and p<0.0001 (****).

The Ca^2+^- CRZ1-independent but synergistic mechanism was also observed when we analyzed the expression of some β-glucosidases coding genes after SCB and cellulose induction (Figure 4A-G, Figure S4 A-F). Regarding β-glucosidases regulation, such mechanism of cooperation was so remarkable that only one of the studied genes *(cebl3b)* was modulated by CRZ1 alone (without Ca^2^+) (Figure 4D). Interestingly, in opposite to the main cellulases of *T. reesei (cel6a* and *cel7a*), β-glucosidases gene control tends to respond more effectively to cellulose stimuli, highlighting the role of these enzymes on plant cell wall deconstruction. It is also worth to notice that β-glucosidases coding genes were a class of genes which we identified a vast number of putative CRZ1 binding sites in our *in silico* analysis, providing us an hypoth esis of why these genes are more responsible to cellulose than other known strongly expressed genes (cellobiohydrolases).

**Figure 5:**
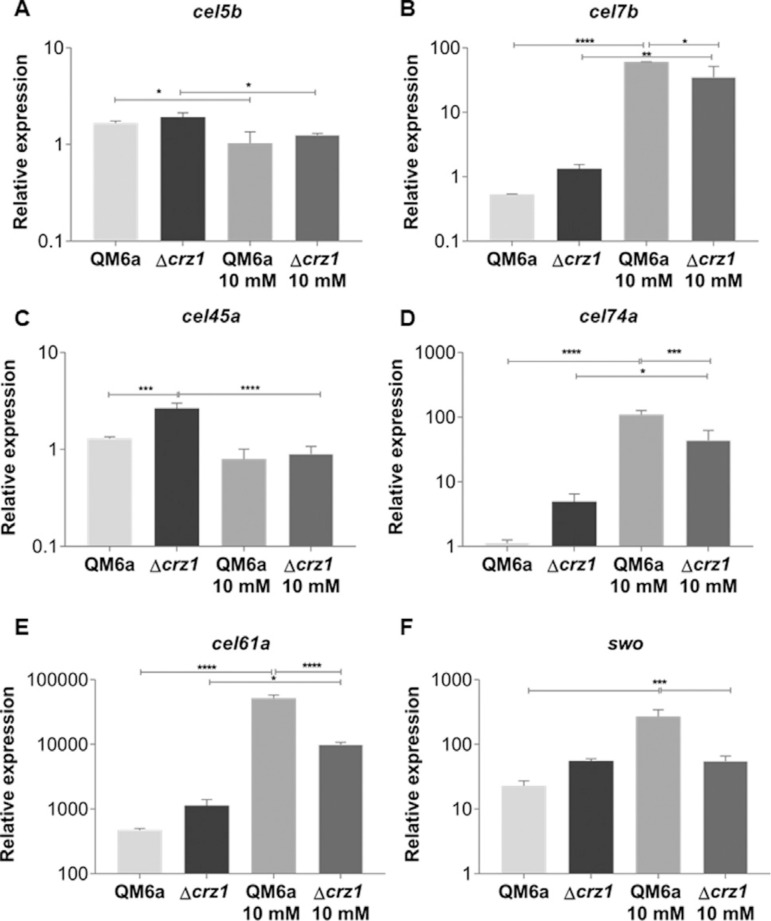
Analysis of differential expression of the endoglucanases (A-C), xyloglucanase (D), LPMO CEL61A (E) and swollenin (F) from QM6a and Δcrz1 T. reesei in the presence of SCB and SCB supplemented with 10 mM Ca^2^+ after 8h of induction. Expression values are represented as log10 means of three biological replicates with standard deviation normalized by glycerol expression levels at the same condition. Statistical significance is represented as asterisks, considering p-value as <0.05 (*), <0.01 (**), <0.001 (***) and p<0.0001 (****).

The most heterogeneous effect on holocellulases expression was noticed for glucanase coding genes expressed in SCB, as shown by Figure 5A-D. In this condition, we observed a Ca^2^+-CRZ1-independent decrease on endoglucanases expression (Figure 5A), a synergistic Ca^2+^- CRZ1-independent effect which resulted in a increase on expression (Figure 5B, D) and finally, a positive modulation exclusively mediated by CRZ1 which loses its potential in a Ca^2+^-synergic mechanism (Figure 5C). Interestingly, after cellulose induction, the Ca^2+^-CRZ1-independent mechanism was capable to potentialize the expression of only *cel7b* gene (Figure S5 B).

The effect of Ca^2^+ and the deletion of *crz1* on accessory proteins expression was also investigated in our study, as shown for the lytic polysaccharide monooxygenases (LPMO) *cel61a* and *swo* (swollenin, an expansin-like protein) genes. In this way, we also observed the effect of a synergistic Ca^2+^-CRZ1-independent mechanism that may positively regulate the expression of these genes in both SCB (Figure 5E-F) and cellulose induction (Figure S5 E-F), highlighting the role of both CRZ1 and Ca^2+^ on the regulation of accessory proteins required for lignocellulose deconstruction.

### Calcium and sugar transporters are regulated by CRZ1 in the presence of Ca^2+^

**Figure 6:**
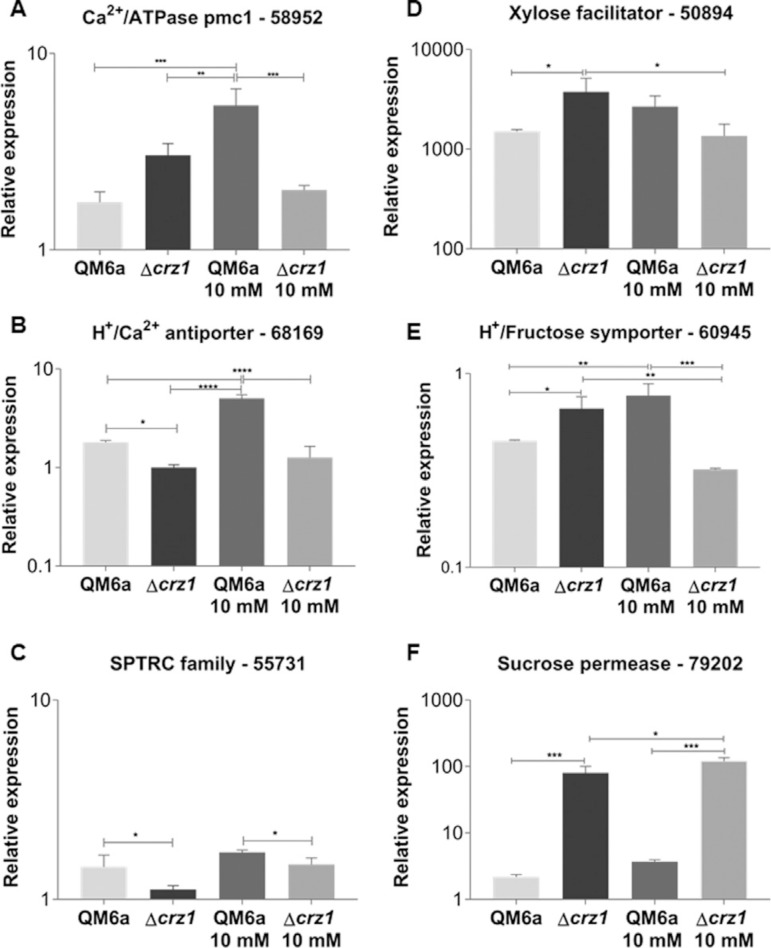
Ca^2+^+ transporter genes (A-C) and sugar transporter (D-F) differential genic expression in the QM6a and Δcrz1 T. reesei strains after 8 h of growing in SCB and SCB supplemented with 10 mM Ca^2+^. Results are expressed as log_10_ means of three biological replicates with standard deviation normalized by glycerol expression levels at the same condition. Statistical significance is represented as asterisks, considering p-value as <0.05 (*), <0.01 (**), <0.001 (***) and p<0.0001 (****). Protein ID of the evaluated genes are supported at *T. reesei* genome database (https://genome.jgi.doe.gov/pages/search-for-genes.jsf?organism=Trire2 https://genome.jgi.doe.gov/pages/search-for-genes.jsf?organism=Trire2).

Since the role of Ca^2+^ is clearly related to the CRZ1 regulatory role in *T. reesei* (Chen *et al*., 2016), we decided to investigate whether calcium transporters were target of modulation by CRZ1. After both SCB and cellulose growth, we observed that the deletion of *crz1* elicited a loss in transcription of these genes in the presence of SCB (Figure 6B-C) while it boosted the expression of the same genes after cellulose induction (Figure S6 B-D). In spite of CRZ1-independent modulator effect, it is also worth to notice that Ca^2+^ synergistically to this TF lacking were able to cause a decrease on the expression of such genes predominantly in SCB in comparison to cellulose (Figure 6A-C, Figure S6 B). Interestingly, our *in silico* analysis also evidenced the occurrence of putative CRZ1 binding sites in promoter regions of these genes, suggesting that calcium homeostasis is precisely regulated at genomic levels by CRZ1, mainly under stress conditions.

Finally, once we observed that carbon source could influence the transcription of holocellulases genes in *T. reesei* in Δ*crz1* strain, we studied the effects of *crz1* knockout on the expression of sugar transporter genes both after SCB and cellulose induction. Interestingly, our results showed that CRZ1 itself plays a regulatory role on the expression of these genes in the presence of complex substrates, since we detected a significant decrease on their expression in the wild type strain in comparison to the mutant strain both grown in SCB (Figure 6D-F). At the same conditions, we also observed a double effect on the synergic Ca^2+^- CRZ1-independent mechanism. In this sense, Ca^2+^ allied to CRZ1 absence, were able to significantly decrease the expression of *Trire_50894* and *Trire_60945* genes, while the opposite effect was achieved by the increase of *Trire_79202* gene expression. In contrast, concerning cellulose-induced differential regulation, we did not detect any differences on expression of the evaluated genes (Figure S7 C-E), suggesting a remarkable role of CRZ1 in the regulation of genes involved in biomass transport.

### Effect of *crz1* deletion on enzymatic activity of holocellulases

**Figure 7:**
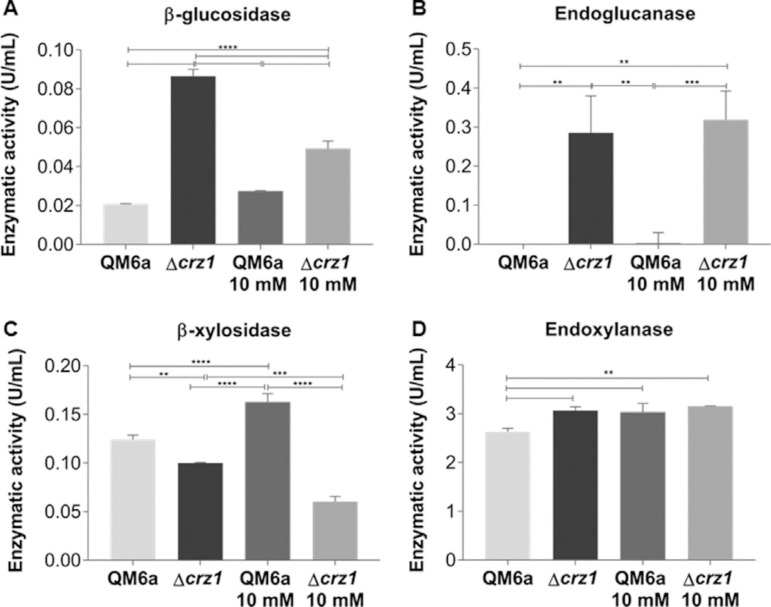
Enzymatic activity of β-glucosidase (A), endoglucanase (B), β-xylosidase (C) and endoxylanase (D) from QM6a and Δcrz1 T. reesei strains supernatants after 8 h of growing in SCB and SCB supplemented with 10 mM Ca^2+^. Results are represented as absolute values in units per milliliter and are representative of a mean of three biological replicates with standard deviation. Statistical significance is represented as asterisks, considering p-value as <0.05 (*), <0.01 (**), <0.001 (***) and p<0.0001 (****).

The analysis of the culture supernatants where the QM6a wild type and *Δcrz1* strains were grown showed that the activity of cellulases (β-glucosidases and endoglucanases) was always superior in the supernatants from Δ*crz1* strain in comparison to the QM6a strain, regardless the carbon source that we evaluated (Figure 7A-B, Figure S8 A-B). Ca^2+^ supplementation in the absence of CRZ1 was able to influence the enzymatic activity of the tested supernatants, mainly for endoglucanases, as we were not able to detect any enzymatic activity in the QM6a strain even in the presence of 10 mM Ca^2+^ for both carbon sources that we investigated.

For xylanase activities (β-xylosidases and endoxylanases) in SCB and cellulose (Figure 7C-D, Figure S8 C-D), we observed that in the Δ*crz1* supernatants the activities were higher compared to the QM6a strain except for the β-xylosidase in SCB (Figure 7C). Interestingly, the enzymatic activities of supernatants whose QM6a strains were grown after Ca^2+^ addition were higher in presence of SCB (Figure 7C-D) and presented a decrease in cellulose induction in comparison to SCB supernatants (Figure S8 C-D). For instance, the *crz1* knockout resulted in supernatants with lower β-xylosidase activity in SCB presence (Figure 7C) and higher xylanases activities measures after cellulose induction (Figure S8 C-D) when compared to the QM6a strain at the same condition.

## Discussion

In this work, we investigated the role of the CRZ1 regulator over the expression of holocellulases and sugar and Ca^2+^ transporters of the WT *T. reesei* QM6a strain. CRZ1 is an important regulator involved in several cellular functions in cell and Ca^2+^/CRZ1-Calcineurin/Calmodulin is one of the best studied signaling pathways which provides protection and sensibility for fungal cells against stress conditions. This signaling pathway is already reported to confer Ca^2+^ and cell wall stress tolerance in *Aspergillus* (Shwab *et al*., 2019), pH responsiveness in *S. cerevisiae* (Roque *et al*., 2016), and it is related to *C. neoformans* viability (Lev *et al*., 2012; Chow *et al*., 2017). In addition, it is also reported that CRZ1 may influence regulatory mechanisms that control holocellulases expression in *T. reesei* (Chen *et al*., 2016).

In this study, we performed bioinformatics approaches to search for putative CRZ1 binding sites in promoter regions of holocellulases, Ca^2+^ and sugar transporter coding genes in *T. reesei* QM6a. This approach was achieved considering the sequences in promoter regions of genes whose differential expression was previously reported (Castro *et al*., 2016). Here we identified that these genes harbored sense and antisense CRZ1 putative binding sites at their ATG upstream sequences and it supports the evidence of the regulation played by this TF independently of carbon source availability. To be clear that CRZ1 was involved in gene regulation in the wild type strain of *T. reesei,* our study reports that holocellulases genes were directly or indirectly modulated by this protein, corroborating the occurrence of putative CRZ1 binding sites sequences in the *T. reesei* genome.

Chen *et al*. (2016) reported the influence of CRZ1 and Ca^2+^ on the transcription regulation of some relevant vegetal biomass deconstructing enzymes in the industrial strain *T. reesei* RUT-C30. This seminal work provided the first evidence that the Calcineurin-CRZ1 pathway was related to holocellulases production in this species. However, it was not elucidated whether the other holocellulases genes were target of regulation in this pathway, as this study was performed using the RUT-C30 industrial strain of *T. reesei* (which does not harness CRE1, the master regulator of CCR in this fungus). Since *T. reesei* is gifted with a vast repertoire of such enzymes, this motivated us to investigate the influence of CRZ1 and Ca^2+^ on the expression of the reminiscent genes, whose expression had not been investigated yet. Therefore, we also were interested in study if the supplementation of complex carbon sources could trigger a differential pattern of sugar transporters expression due to the high amount of distinct sugars present in SCB culture supernatant.

**Figure 8:**
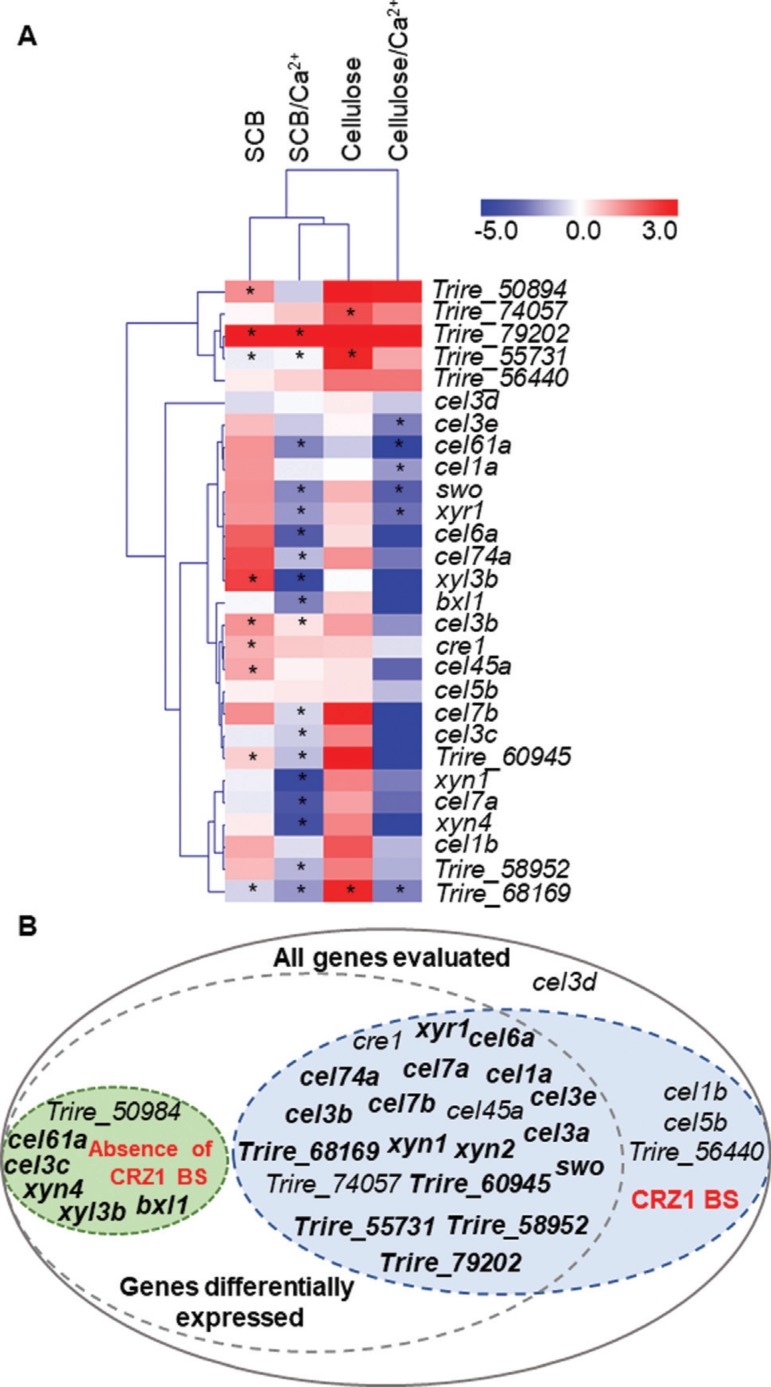
(A) Heatmap of the differential expression profile of the holocellulases and calcium and transporter sugars after 8 h of induction in the presence or in the absence of 10 mM Ca^2^+. Values are representative of the expression obtained with mutant *crz1* strain related to the QM6a strain. Genes presenting representative statistical significance are show with asterisks. ID codes for calcium and sugar transporters are supported in *T. reesei* genome database (https://genome.jgi.doe.gov/Trire2/Trire2.home.htm). (B) Representative scheme of genes differentially expressed in the absence of CRZ1 in *T. reesei* and its relation to the occurrence or absence of binding sites (BS) for CRZ1 at genomic level. Bold characters represent the genes whose expression was modulated by 10 mM Ca^2+^ addition.

In this sense, we observed that distinct classes of holocellulases were differentially expressed regardless the fungal growth condition. Although cellulose was not capable to modulate a major number of genes independent of CRZ1 presence, SCB provided the major number of differentially expressed genes after its induction process (Figure 8A). This inducer capacity of SCB over the industrial strain RUT-C30 of *T. reesei* was already reported (Borin *et al*., 2015). In their study, Borin *et al*. (2015) found 24 biomass degrading enzymes in *T. reesei’s* RUT-C30 secretome after SCB exposure, showing that this carbon source is a potential activator of holocellulases production in this fungus. Interestingly, in our work, SCB induction was able to elicit a higher holocellulases transcriptional response in comparison to cellulose (Figure 8A).

We also observed a major number of differentially expressed genes in the presence of SCB supplemented with Ca^2^+ in the comparison to cellulose exposure at the same Ca^2^+ concentration (Figure 8A), endorsing the previously reported effect of carbon source on modulation of *T. reesei* transcriptional profile (Castro *et al*., 2014b). We observed that Ca^2^+ seems to be critical for the expression of many holocellulases genes more directly than the carbon source whose fungal growth was previously established, emphasizing its crucial role on transcription of these genes. These evidences strongly confirm the synergistic effect of Ca^2+^ on the expression of well-studied holocellulases (Chen *et al*., 2016) and highlight new findings observed for holocellulases genes which had not been studied yet (here described).

Ca^2+^ plays a regulatory role on several mechanisms in *T. reesei,* e.g. on protein secretion (Mach *et al*., 1998) and on events related to the CRZ1-Calcineurin pathway (Chen *et al*., 2016). In this sense, Chen *et al*. (2016) reported the influence of 5 mM Ca^2+^ on the transcription of relevant industrial genes of *T. reesei* RUT-C30, such as *cbh1* and *xyr1.* In our study, 10 mM Ca^2+^ was able to potentialize the expression of *xyr1* at the first 8 h of induction. Our results differ from previous findings reported by Chen *et al*. (2016) since we used the double of Ca^2+^ concentration and a longer induction time than the former work (whose study showed the first 6 h of cellulose exposure as inefficient to increase expression of *xyr1* in the industrial strain of *T. reesei).* Taking this advantage, here we suggest that a higher Ca^2+^ concentration might elicit a faster gene activation in the presence of cellulose. Interestingly, we also observed a massive loss in transcription of *xyr1* in our Δ*crz1* strain in the presence of Ca^2^+, in accordance with the results obtained by Chen *et al*. (2016).

In the face of the character of regulatory proteins, our results clearly showed that CRZ1 may act as a bifunctional regulator, assuming different profiles both in activation or in the repression of holocellulases in the wild type strain of *T. reesei,* not only playing a specific activating or repressing role over gene expression. We consider this a relevant characteristic for the engineering of *T. reesei,* especially when taking the advantage of the use of SCB as an inducer of holocellulases gene expression, as SCB is an agroindustrial residue that can be widely employed for biofuel production, especially in Brazil, which leads sugarcane bagasse production worldwide.

Our results also show that CRZ1-mediated regulation was not limited to lignocellulose degrading enzymes coding genes, but also intersected the regulatory network that coordinates holocellulases expression in *T. reesei* QM6a. Therefore, we report that CRZ1 can modulate the transcript levels of relevant TFs such as XYR1 and CRE1 (inactive in RUT-C30 background). The relevance of CRE1 repression is also target of discussion of *T. reesei* regulation. Here we found that CRZ1 positively modulates the expression of CRE1, suggesting that CRZ1 may synergistically contribute to the modulation of holocellulases expression. For instance, we reiterate that the results which we obtained are related to the presence of CRE1 in the WT *T. reesei* genome, suggesting that different perspectives could be achieved with deeper studies highlighting the interaction between CRZ1 and CRE1 (or its lack).

Albeit the effect of SCB on enzyme expression, Ca^2+^ and sugar transporter proteins had already been reported in a *T. reesei* RUT-C30 gene co-expression network study by Borin *et al*. (2018), here we firstly described the seminal importance of Ca^2+^ on the expression of sugar transporters in a CRZ1 synergistic context in the *T. reesei* QM6a strain. According to our data, we hypothesize that under Ca^2+^ exposure, *T. reesei* QM6a may coordinate the transcription of sugar transporters to keep metabolic homeostasis through the Ca^2+^-CRZ1/Calcineurin pathway. This is particularly interesting when the expression pattern was significantly modulated in the presence of SCB, suggesting that complex sugars could induce a fine mechanism of regulation in order to balance the uptake of carbohydrates in the presence of complex sugar-enriched medium.

In addition, our results indicate that the synergic effect of CRZ1 and Ca^2^+ was also relevant for the regulation of Ca^2^+ transporters in our study strain. We were able to detect not only the influence of such cooperative effect, but also the carbon-source dependence modulation effect over the transcription of such genes. By this we mean that Ca^2^+ can potentialize the transcription of Ca^2+^ transporters in a carbon dependent availability. In summary, we hypothesize that the differential expression of sugar transporters could modulate the uptake of sugars, which in turn, could influence the expression of CRZ1, that, in the presence of Ca^2^+, could elicit a response to increase the number of Ca^2+^ transporters to keep cellular homeostasis.

Enzymatic activity of culture supernatants was also widely variable in the distinct strains that we evaluate. We attribute this to the bimodal character of CRZ1, as it could directly or indirectly modulate transcription of enzyme genes. Even though a relation between transcription and translation is not always direct, the activity of β-xylosidases is significantly reduced in the mutant strain of *crz1* in the presence of Ca^2+^, suggesting that the synergic effect CRZ1-Ca^2+^ may be suggestive of an indirect mechanism of regulation on the activity of these enzymes. Additionally, it is important to consider that Ca^2+^ could modulate the enzymatic activity of supernatant enzymes and this relation is a deep layer of biochemical analysis in any study.

The effect of sense or antisense CRZ1 putative binding sites in promoter regions is one of the main evidences that supports regulation in the QM6a strain of *T. reesei.* The effect of arrangements and repetitions of TFs binding sites in *T. reesei* was already reported by Kiesenhofer *et al*. (2018) as an important parameter for engineering transcriptional regulation in this fungus. As observed in our work, through an *in silico* analysis, from the total set of differentially expressed genes, only 6 of them did not harness CRZ1 putative binding sites and only one of these was not modulated by Ca^2+^ (Figure 8B). These evidences strongly suggest that the synergistic mechanism played by CRZ1 and Ca^2+^ is required for holocellulases and transporter coding genes expression in *T. reesei* QM6a even not in the presence of stress conditions. In addition, our data also assert that CRZ1 activation is pivotal for gene regulation, but Ca^2+^ is determinant for an increasing modulation (positive or negative) on gene expression levels, emphasizing the necessity of deeper studies on indirect holocellulases regulation in *T. reesei.*

Finally, we summarize the importance of investigating CRZ1 role on the expression of holocellulases in *T. reesei.* Deeper studies are necessary to a fully understand the network comprising CRZ1 and Ca^2+^, and according to our results, we also suggest that there is may be another unknown regulatory pathway directly or indirectly responsive to Ca^2+^ and this could influence CRZ1 regulatory role in an indirect manner.

## Conclusions

In this study, we used bioinformatics approaches to search for putative TFs in relevant industrial genes of *T. reesei*. Here we demonstrate the huge potential of *in silico* methods to explore transcription regulation in important enzyme biofactories, mainly in industrial filamentous species. In summary, we describe the construction of a *T. reesei crz1* knockout strain through bioinformatics prediction and validation of the regulatory role of the bifunctional CRZ1 protein on transcriptional regulation of *T. reesei.* Allied to Ca^2+^, an important modulator of the regulatory effects of CRZ1, holocellulases and calcium/sugar transporter genes can be positively or negatively modulated in a CRZ1- synergistic mechanism, as well as the enzymatic activity of such enzymes may direct or indirectly be affected by CRZ1 regulatory role. These phenomena are suitable attributes to biotechnological industry applications, highlighting fungi engineering as a fundamental platform to enzyme production at large scale.

## Supplementary material

The following online material is available for this article:

Table S1 - Primers used in this study for the construction of the crz1 deletion cassete.

Table S2 - Primers used in this study for real time PCR.

Figure S1 - Molecular procedures to confirm crz1 ORF deletion from *T. reesei* QM6a genome.

Figure S2 - Expression profile of regulators and main cellulase from *T. reesei*.

Figure S3 - Expression of holocellulases in response to Avicel.

Figure S4 - Expression of P-glucosidases genes of *T. reesei.*

Figure S5 - Expression of endoglucanases and accessory proteins.

Figure S6 - Expression of transporter genes.

Figure S7 - Expression of transporters in response to SCB and Avicel.

Figure S8 - Expression of additional holocellulases in response to Avicel.
