# Supplementary material for: CRZ1 regulator and calcium cooperatively modulate holocellulases gene expression in Trichoderma reesei QM6a
Source: Genet Mol Biol. 2020 May 8;43(2):e20190244. doi: 10.1590/1678-4685-GMB-2019-0244 (PMC7212764; doi:10.1590/1678-4685-GMB-2019-0244)
Supplement: Supplementary file 1 [file 1415-4757-GMB-43-2-e20190244-s1.pdf]

Genetics and Molecular Biology 43, 2 (2020)

Supplementary Material to "CRZ1 regulator and calcium cooperatively modulate holocellulases gene expression in *Trichoderma reesei* QM6a"

Table S1 - Primers used in this study for the construction of the *crz1* deletion cassete.

PRIMER	SEQUENCE
5' Pcrz1	CCAGTCCTGTTTCGCCATGTACGC
3' Pcrz1-pyr4	CCCAGACAAGACAAGGCAAGGAGGATGTGTCAAATAGGGCTGG
5' pcrz1 -pyr4	CCAGCCCTATTTGACACATCCTCCTTGCCTTGTCTTGTCTGGGTTCCT
3' Pyr4 - Tcrz1	TGCGACAACCCATCTAATCGGTACGGTTGATTGTTGCCGTCCGTTTC
5' pyr4-Tcrz1	GAAACGGACGGCAACAATCAACCGTACCGATTAGATGGGTTGTCGCA
3' Tcrz1	GCCATCCACCCCGAACTTCACAA
5' Pcrz1 -pRS426	GTAACGCCAGGGTTTTCCCAGTCACGACGCCAGTCCTGTTTCGCCATGTACGC
3' Tcrz1 - pRS426	GCGGATAACAATTTCACACAGGAAACAGCGCCATCCACCCCGAACTTCACAA
5' Pcrz1 check1	ATCCATACTACGGACCTTGTGCG
3' pyr4 check1	GTGTACTGCAGCTCGACGGTGT
5' pyr4 check 2	ATGCCTTTATCCACATGACGCCCG
3' Tcrz1 check 2	GCAATCAAAAGTCGCAGAAGACCG
PF crz1 qRT-PCR	CCCAAGAGATTCACCAGAGC
PR crz1 qRT-PCR	TTTCCTGTCATGCTGTCGAG
5' crz1 ORF	TAAACCAATCCCACTCGCCG
3' crz1 ORF	GCTCTGGTGAATCTCTTGGGAC
