# Supplementary material for: CRZ1 regulator and calcium cooperatively modulate holocellulases gene expression in Trichoderma reesei QM6a
Source: Genet Mol Biol. 2020 May 8;43(2):e20190244. doi: 10.1590/1678-4685-GMB-2019-0244 (PMC7212764; doi:10.1590/1678-4685-GMB-2019-0244)
Supplement: Supplementary file 2 [file 1415-4757-GMB-43-2-e20190244-s3.pdf]

Supplementary Material to "CRZ1 regulator and calcium cooperatively modulate holocellulases gene expression in *Trichoderma reesei* QM6a"

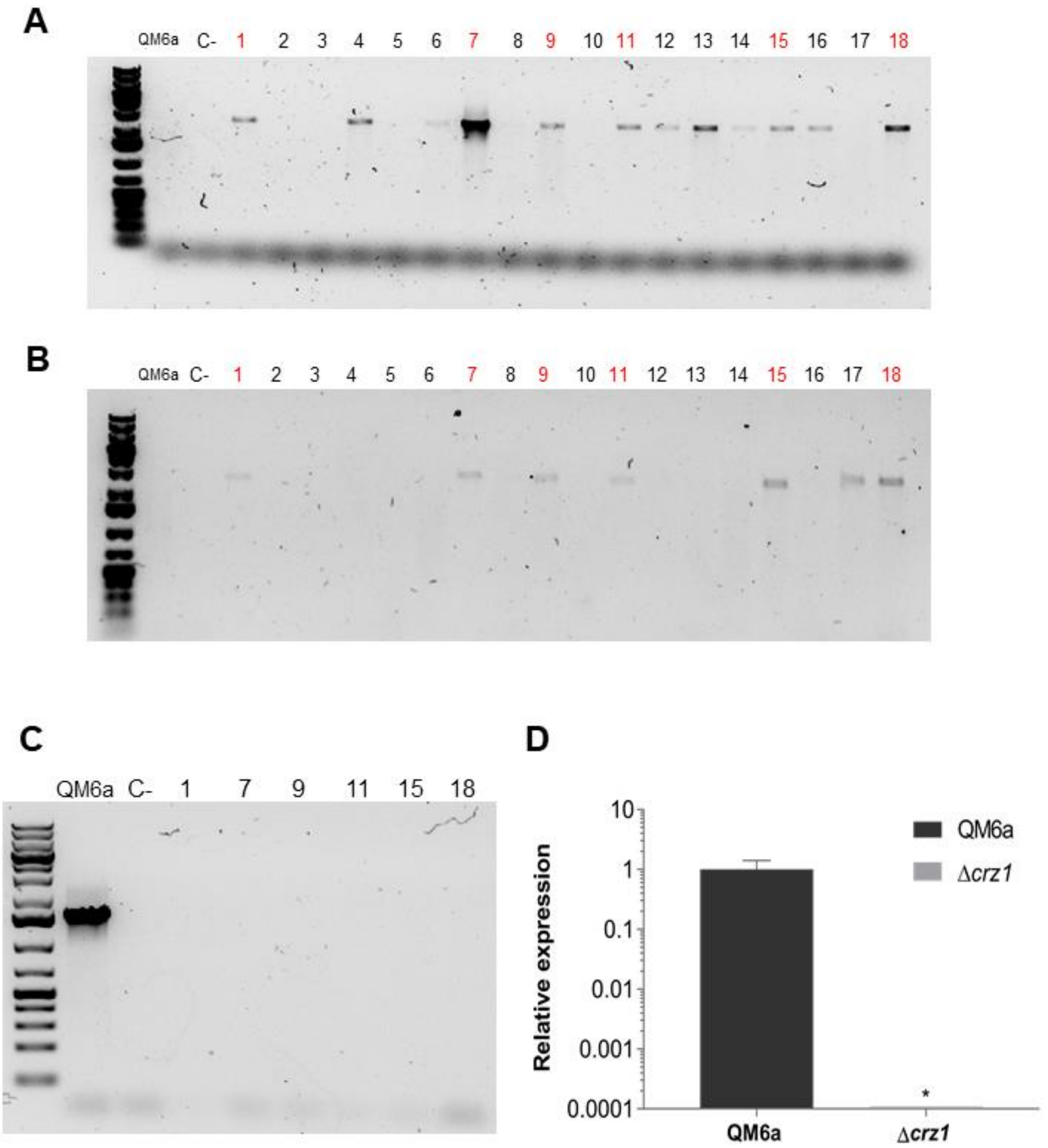

Figure S1 - Molecular procedures to confirm *crz1* ORF deletion from *T. reesei* QM6a genome. Numbers in lanes represent the numeric distribution of transformants obtained with the recombination. **A-** PCR whose amplicon of 2.6 kb comprises the annealing of a promoter external region primer and *pyr4* selectable marker. **B** – PCR to confirm the correct integration in *T. reesei* genome whose amplicon of 2.5 kb comprises the amplification of *pyr4* region with an external terminator sequence beyond the cassette of integration. **C** – Null amplification of the *crz1* ORF using a conventional PCR in the positive transformants previously obtained (**A-B**). **D-** Quantitative PCR of the *crz1* in the mutant strain obtained with homologous recombination.
