# Supplementary material for: CRZ1 regulator and calcium cooperatively modulate holocellulases gene expression in Trichoderma reesei QM6a
Source: Genet Mol Biol. 2020 May 8;43(2):e20190244. doi: 10.1590/1678-4685-GMB-2019-0244 (PMC7212764; doi:10.1590/1678-4685-GMB-2019-0244)
Supplement: Supplementary file 3 [file 1415-4757-GMB-43-2-e20190244-s2.pdf]

Supplementary Material to "CRZ1 regulator and calcium cooperatively modulate holocellulases gene expression in *Trichoderma reesei* QM6a"

Table S2 - Primers used in this study to quantify the differential expression levels of the holocellulases and calcium and sugar transporter genes in the *T. reesei Δcrz1* strain that we obtained.

Holocellulases and swollenin primers

Name	Protein ID	Sequence
cel6a R	72567	TGT TCC ACC CGT TGT AGT TG
cel6a F		ACA AGA ATG CAT CGT CTC CG
cel3a R	76672	TAG CTG AGA TCT CGT CGT C
cel3a F		CTG TAC ATC ACC TAC CCA TC
cel7a R	123989	GGT AGC CTT CTT GAC TGA GT
cel7a F		CCG AGC TTG GTA GTT ACT CTG
cel1a R	120749	AAT CAG CTC GTC AAA CAG CG
cel1a F		TTT GCC TGG TCG CTC ATG
cel61a R	73643	ACC GCT GCC ACC ACA CTG
cel61a F		GCG CCA CTG TTC CTG GAG
cel45a R	49976	TGG TCC AGA ATG CAC TCG
cel45a F		CAG CGA CGT CTA CAT TGG
cel7b R	122081	AGG TCT TGG AGG TGT CAA CG
cel7b F		CCC TCA ACA CTA GCC ACC AG
cel1b R	22197	TCC AAG TGC GAG TCA AAG TAG
cel1b F		CCA TCT ACA TCA CCG AGA ACG
cel3b R	121735	ATG TGG AGG TTG GAG AAC TTG
cel3b F		CCA GGA TAA CTT CAA CGA GGG
cel3c R	82227	ATC CCA ACC CCA TTC CTT TC
cel3c F		GCG TAC AAT GGC ATC AAT GG
cel3e R	76227	TCG TGA GTC CAA AGT GAA CAG
cel3e F		ATG TCT GGA AGT GAG GTT GC
cel74a R	49081	TGA TGT CTT TCC AAG TTC CCC
cel74a F		GCC TTG TAT CTG ACC TAT TCC G
xyn1 R	74223	TCT GTC TTT TGG GCT TGG AG
xyn1 F		GGC CAA ATT ATC GTC AAC TGT C
xyn2 R	123818	TCT GCA CAG TAA CAG TTC CG
xyn2 F		TGT CAA CGA GCC TTC CAT C
xyn4 R	111489	GAC AGG TTG GCA AAA TGG TG
xyn4 F		TGT CAG CAA TTC GGG TCT TC
cel3c F	82227	GCG TAC AAT GGC ATC AAT GG
cel3c R		ATC CCA ACC CCA TTC CTT TC
bxl1 F	121127	CAA GTC TGG AAT GAG GCT CTG
*bxl1*R		TGA TGT CGG CAA TCT GGT G
bxl3 F	58450	TCA ATG TTC CTC TCC ATG CC
*bxl3*R		GGT TGG AGA TGG AGT AGA TGT TG
swo F	123992	CCA AAC TAT ACG AGT AGC C
swo R		GAG TGA ATG TCT TGA TGG
Actin F	44504	TGA GAG CGG TGG TAT CCA CG
Actin R		GGT ACC ACC AGA CAT GAC AAT GTT

Transcription factor primers

Name	Protein ID	Sequence
xyr1 F	122208	CAA TCC TCT CCG TCG CTA TTC
xyr1 R		CTG TTG CCG AAT GTG TTG AC
cre1 F	120117	CTC CTA CTC GTC CTT TGT CAT G
cre1 R		GCA AGC ATC GTA ATG TCG TTG
crz1 F	36391	CCC AAG AGA TTC ACC AGA GC
crz1 R		TTT CCT GTC ATG CTG TCG AG

Calcium and sugar transporter primers

Name	Protein ID	Sequence
Trire_58952 F	58952	CTC TCC AAA TGC TAC CCT TAC C
Trire_58952 R		CCG ACT ACA TCC GCC ATG
Trire_55731 F	55731	ACG AAT CTA CCC TTT GCT GG
Trire_55731 R		CGT AGT CGG TTG TCC TTC ATC
Trire_56440 F	56440	TGA GTG AAT GGA TAG ATG CCG
Trire_56440 F		AGA GGG TGC CAA TGA GAA TC
Trire_74057 F	74057	CGT CAT GGG TGT TCT ATA CTC G
Trire_74057 R		AAT TGC TCC CAC TCT TCC AC
Trire_68169 F	68169	TCT GGT TCA ACT TCC TCG C
Trire_68169 R		CAC TCA TAG GAC CCA ACA CG
Trire_50894 F	50894	TCG TCT TTC GTC TCA TTG TCG
Trire_50894 R		GAT TGC CTG TTT GTC TTC CG
Trire_79202 F	79202	GAG TAC CTG GTC AAC GTG AG
Trire_79202 R		CAA ACC TAT GTG TTG GCT CAG
Trire_60945 F	60945	GTC TCT GGG CGT GTT ATT CTC
Trire_60945 R		CTT CTC CAA GCG CAA TGT TG
