# Supplementary material for: CRZ1 regulator and calcium cooperatively modulate holocellulases gene expression in Trichoderma reesei QM6a
Source: Genet Mol Biol. 2020 May 8;43(2):e20190244. doi: 10.1590/1678-4685-GMB-2019-0244 (PMC7212764; doi:10.1590/1678-4685-GMB-2019-0244)
Supplement: Supplementary file 8 [file 1415-4757-GMB-43-2-e20190244-s8.pdf]

Supplementary Material to "CRZ1 regulator and calcium cooperatively modulate holocellulases gene expression in *Trichoderma reesei* QM6a"

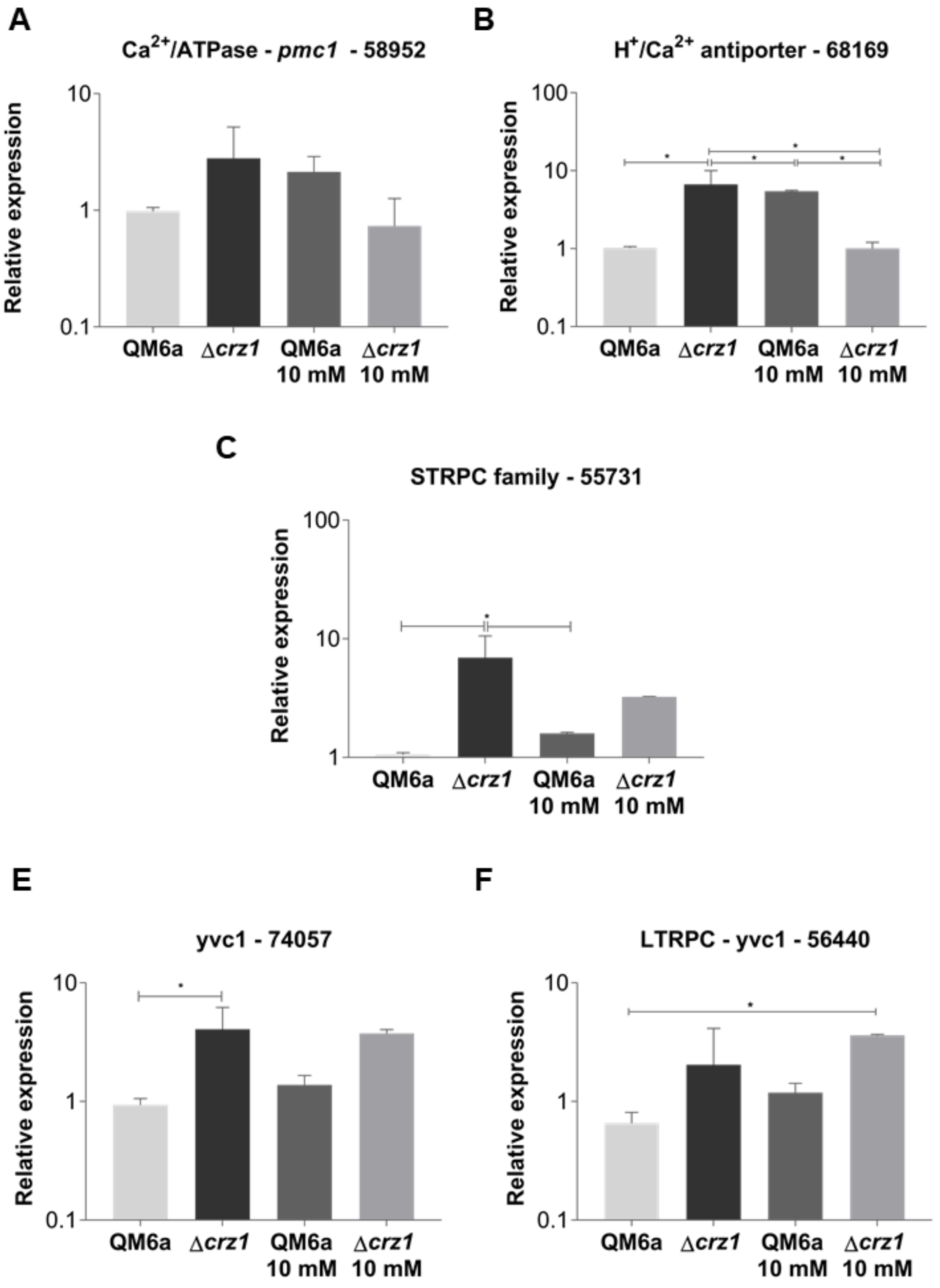

Figure S6 - A-E – qRT-PCR results for differential expression analysis of the Calcium-transporter genes differential expression in the QM6a and *Δcrz1 T. reesei* strains after 8 h of growing in commercial cellulose (Avicel – Sigma Aldrich®) supplemented or not with 10 mM Ca^{2+}. Expression values are represented as $\log_{10}$ means of three biological replicates with standard deviation normalized by glycerol expression levels at the same condition. Statistical significance is represented as asterisks, considering p-value as at least < 0.05 (* < 0.05 < ** < 0.005 < *** < 0.0001 < ****). Protein ID of the evaluated genes are available at *T. reesei* genome database (https://genome.jgi.doe.gov/pages/search-for-genes.jsf?organism=Trire2).
