# Supplementary material for: CRZ1 regulator and calcium cooperatively modulate holocellulases gene expression in Trichoderma reesei QM6a
Source: Genet Mol Biol. 2020 May 8;43(2):e20190244. doi: 10.1590/1678-4685-GMB-2019-0244 (PMC7212764; doi:10.1590/1678-4685-GMB-2019-0244)
Supplement: Supplementary file 10 [file 1415-4757-GMB-43-2-e20190244-s10.pdf]

Supplementary Material to "CRZ1 regulator and calcium cooperatively modulate holocellulases gene expression in *Trichoderma reesei* QM6a"

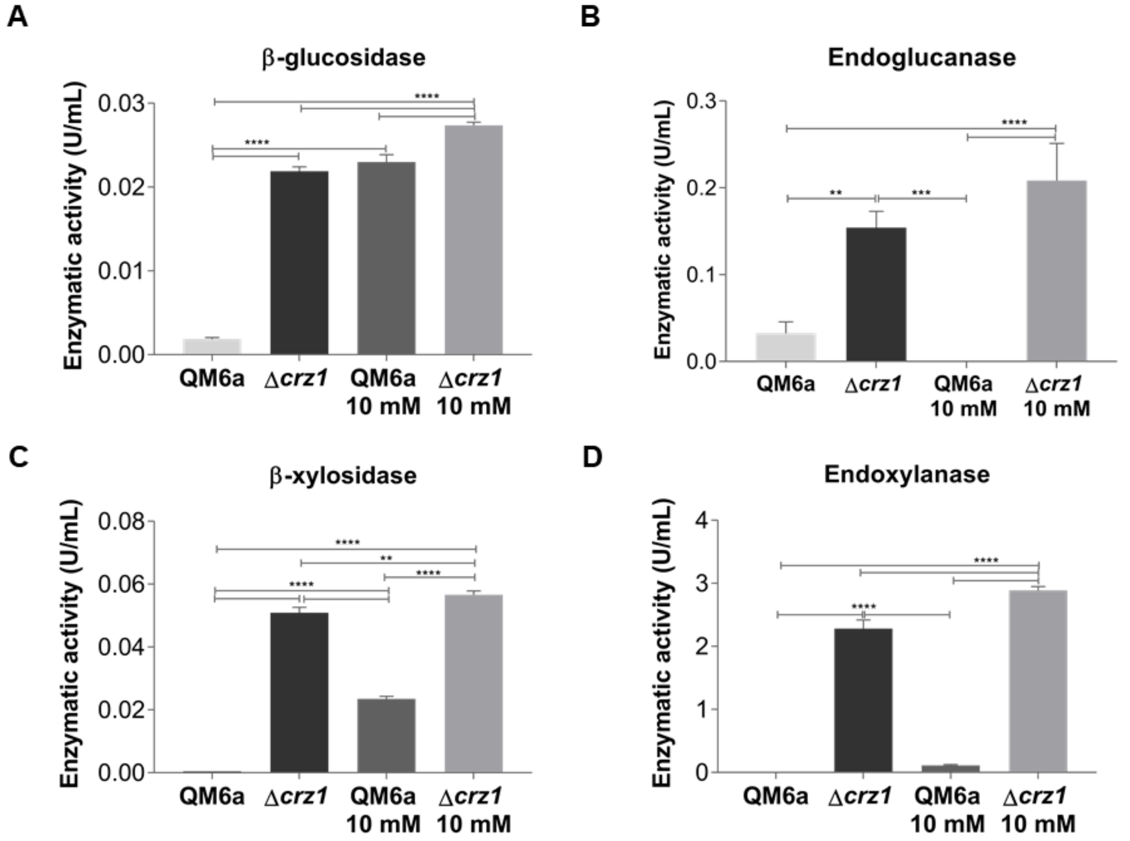

Figure S8 - Enzymatic activity measurements for β-glucosidase (**A**), endoglucanase (**B**), β-xylosidase (**C**) and endoxylanase (**D**) from QM6a and *Δcrz1 T. reesei* strains supernatants after 8 h of induction with commercial cellulose (Avicel – Sigma Aldrich®) supplemented or not with 10 mM Ca^{2+}. Results are represented as absolute values in unities per milliliter and are representative of a mean of three biological replicates with standard deviation. Statistical significance is represented as asterisks, considering p-value as at least < 0.05 (* < 0.05 < ** < 0.005 < *** < 0.0001 < ****).
